# Covid-19 as an issue of memory, truth, and justice: an interview with Deisy Ventura

**DOI:** 10.1590/S0104-59702023000100053

**Published:** 2023-10-23

**Authors:** Deisy Ventura, Carlos Henrique Assunção Paiva, Claudia Agostoni, Vivian Mannheimer, Marcos Cueto

**Affiliations:** i Professora titular, Faculdade de Saúde Pública/Universidade de São Paulo. São Paulo – SP – Brasil deisy.ventura@usp.br; ii Pesquisador do Observatório História e Saúde, professor do Programa de Pós-graduação em História das Ciências e da Saúde, Casa de Oswaldo Cruz/Fundação Oswaldo Cruz. Rio de Janeiro – RJ – Brasil carlos.paiva@fiocruz.br; iii Pesquisadora titular, Instituto de Investigaciones Históricas/Universidad Nacional Autónoma de México. Cidade do México – México agostoni@unam.mx; iv Doutora pelo Programa de Pós-graduação em Comunicação/Pontifícia Universidade Católica do Rio de Janeiro. Rio de Janeiro – RJ – Brasil vmannheimer@gmail.com; v Editor científico, pesquisador, Casa de Oswaldo Cruz/Fiocruz. Rio de Janeiro – RJ – Brasil marcos.cueto@fiocruz.br

**Keywords:** Covid-19, Global health, Human rights, Memory, Covid-19, Saúde global, Direitos humanos, Memória

## Abstract

This interview with Deisy Ventura, professor at the Faculty of Public Health of the Universidade de São Paulo, discusses the political dimension of the covid-19 pandemic in Brazil. She has become a leading reference on the subject due to her extensive knowledge of international law, with a focus on health. In this interview, Deisy Ventura offers some reflections on global health and discusses the handling of the pandemic in Brazil and its human rights implications. According to Ventura, the Brazilian government had a systematic policy for the spread of the virus, and the pandemic should be treated as a matter of memory, truth, and justice.

## Resumo

Entrevista com Deisy Ventura, professora titular da Faculdade de Saúde Pública da Universidade de São Paulo, que discute a dimensão política da pandemia de covid-19 no Brasil. A pesquisadora se tornou uma das principais referências no assunto por seu amplo conhecimento de direito internacional com foco em saúde. Na entrevista, ela apresenta reflexões relacionadas à saúde global, além de discutir a gestão da pandemia no país e suas implicações para os direitos humanos. De acordo com a pesquisadora, houve no Brasil uma política governamental sistemática de disseminação do vírus, e a pandemia deveria ser tratada como uma questão de memória, verdade e justiça.

The criticism and research of Deisy de Freitas de Lima Ventura, professor of ethics at the Faculty of Public Health of the University of São Paulo (Faculdade de Saúde Pública da Universidade de São Paulo, FSP/USP), has become a fundamental reference on the covid-19 pandemic in Brazil. She used evidence to demonstrate the government’s systematic policy to spread the coronavirus in the country. In this interview, Deisy Ventura talks about her career, her inestimable research on global health, human rights, and the covid-19 pandemic, and the essential links between memory, history, and justice. The interview also provides some important considerations for understanding the political dimension of the pandemic in Brazil.

*Marcos Cueto: You’re often contacted by newspapers and magazines to make statements and you also managed to get some critical issues introduced into the public debate at an early stage, like the legal norms produced by the Brazilian government to spread the virus, and crimes against humanity. Could you tell me how you started your research on covid-19 in 2020?*

Perhaps the most important thing to clarify before I go into my research since 2020 is that I have a degree in law, specifically international law. I started my career working with issues related to Latin American integration. I’m from the far south of Brazil. I was born in Santa Maria, a city that’s two hours away from Uruguay, and I always had more cultural affinities with colleagues from Argentina or Uruguay than with ones from São Paulo, Rio de Janeiro, or Brasília. When Mercosur was created, in 1991, the Federal University of Santa Maria (Universidade Federal de Santa Maria), where I had earned my degree, set up a master’s degree in Latin American integration. Working as a professor at the university from 1992 onwards, I began to engage in the process of regional integration. At the time, we believed strongly in Mercosur and in the role Rio Grande do Sul [the southernmost state of Brazil] had to play in it. I started my teaching and research career working on legal aspects of regional integration. That was what prompted me to go to Paris to do a master’s and then a PhD, thinking about what this legal framework for integration could look like, because Europe had made a lot of progress in overcoming the complex legal issues involved in eliminating trade barriers between countries, and in other advanced forms of cooperation. The driving force for my academic career was this interest in regional integration. In 2002, when I came back from France having completed my PhD, I worked for three years at the Mercosur Secretariat in Montevideo as a legal advisor in the Mercosur negotiations. It was there that I met Geraldo Lucchese, a legislative advisor for the Brazilian Chamber of Deputies (Câmara dos Deputados), who at the time was leading the negotiations in the field of health.^1^ Another person there in Montevideo was the late lamented Ana Paula Jucá, who worked at Brazil’s public health surveillance agency, along with several other excellent public health specialists in the region. I approached Geraldo, Ana, and some health negotiators, who were receptive to us, as opposed to the diplomats, who were not. I was one of the first four international employees of Mercosur. Until then, the Secretariat had been manned exclusively by national employees on loan, mainly from Uruguay. After a public competition, which attracted hundreds of applicants, the first officials of the bloc were recruited: the Argentinean jurist Alejandro Perotti, the economists Oscar Stark Robledo (Paraguay) and Marcel Vaillant Alcalde (Uruguay), and myself. We had a lot of trouble dealing with the diplomats, but the health personnel were interested in thinking about the quality and significance of the legal norms, and I gradually fell in love with the issues around health. Obviously, I also worked on other negotiations. For example, I helped draft the regulations for the High-level Meeting on Human Rights in Mercosur. I worked on several fronts, but health was the one that attracted me most. It was at that time that I met Sueli Dallari, one of the pioneers of public health law in Brazil. She was responsible for establishing this discipline and the Center for Public Health Law Research (Centro de Estudos e Pesquisas de Direito Sanitário, Cepedisa), where I work today, together with Fernando Aith, also a full professor, and other researchers here at FSP/USP. I started to coauthor texts with Sueli on topics that involved both integration and health, such as the precautionary principle and public health standards in the context of regional integration (Dallari, Ventura, 2002). I followed this course until I felt I’d said everything I had to say about regional integration law, because for political reasons, the Mercosur project sadly reached an impasse.

When I moved to the University of São Paulo (USP) in 2007, I had already decided I wanted to work with health and international health negotiations. The more I studied the subject, the more fascinated I became by the literature on pandemics. Sueli Dallari was a pivotal figure in this choice, in several ways. In July 2009, we coauthored an article together on A(H1N1) influenza, in which we wrote: “to treat the ongoing flu pandemic as a one-off event would be a big mistake,” and “the deplorable state of global economic inequality also causes an uneven distribution of the burden of public health emergencies. The poor bear the heaviest burden” (Dallari, Ventura, 31 jul. 2009). A few days later, in August 2009, she asked me to stand in for her at an extraordinary session of the Chamber of Deputies to discuss the A(H1N1) influenza pandemic, in the presence of the then minister for Health, José Gomes Temporão. Michel Temer was speaker of the House. Osmar Terra, a “flat earther” (a person who denies scientific evidence) who later played an odious role in Jair Bolsonaro’s parallel cabinet, was the Health secretary of the state of Rio Grande do Sul. Some figures who featured in this pandemic were already there in completely different positions. It was a different time. The minister for Health went to the Chamber of Deputies to talk to the deputies and provide answers, and I took part in the session representing my research center, Cepedisa (FSP/USP). I hadn’t been at USP for long and I was struck not so much by the A(H1N1) influenza per se, because clearly it didn’t have the dimensions of covid-19, but by what could happen inside Congress, as I observed the position each person took. There was one deputy, for example, who proposed banning handshaking in Brazil. It was an extraordinary opportunity to witness first-hand the madness that could kick off in a bigger pandemic. At the same time, Temporão was there, who was very competent. Taking part in that hearing was a decisive experience for me, shaping my perspective on public health crises.

Pandemics became my main research interest. No-one working with the law understood why. My colleagues thought I’d lost my mind, turned into a hippie. It seemed completely far-fetched. I did my habilitation thesis on the A(H1N1) influenza at the Institute of International Relations (Instituto de Relações Internacionais) at USP, where I was teaching at the time and where I now serve as deputy chair. The people working in health showed some interest, but were somewhat diffident because I was a lawyer. Few law colleagues took any interest, but I carried on working. I had other research agendas as well, mainly migration and refugees, always from a human rights perspective. It was precisely because of my interest in the rights of migrants that I kept such close tabs on the literature on global health security. In 2013, the Graduate Program in Global Health and Sustainability was created here at FSP/USP, led by Helena Ribeiro and the late lamented Paulo Fortes. Despite the near indifference of my law colleagues, I found some space for myself in the area of international relations, which gradually took interest in this topic and in public health. In the academic area of public health, there’s still a long way to go for international issues to be recognized as an important topic, but the discussion on emergencies was much more advanced, and the mood was much more receptive at FSP/USP than it was among legal scholars.

In January 2020, I was certainly one of few legal experts dedicated to these topics, and not just in Brazil. When the World Health Organization (WHO) declared an emergency, there was the case of how to rescue the Brazilians in Wuhan, China, which served as an impetus for law n.13.979, which came into force on February 6, 2020 (Brasil, 6 fev. 2020). It wasn’t the case that President Bolsonaro was mature enough to recognize the importance of advanced epidemiological legislation. Initially, he said that he wasn’t going to bring the Brazilians back. This statement had bad repercussions so he decided he would bring them back. Then, the people from the Ministry of Health, including Minister Luiz Henrique Mandetta, said something like: if we bring the Brazilians back and confine them, they’ll sue us and will end up being released, because there’s no specific legislation to justify such confinement. That in turn meant there was a risk people would claim that by repatriating Brazilians from China, the president had let covid-19 into Brazil. This was the thinking behind the bill that the minister of Health sent to Congress. The bill was fast-tracked and took three days to get through Congress because the government wanted to get those Brazilians back quickly from China in a large-scale military and propaganda operation, which the Brazilian Air Force called “Operation Return to the Beloved Homeland Brazil” (Operação Regresso à Pátria Amada Brasil) [reflecting the motto of the Bolsonaro government].

After the WHO adopted the International Health Regulations in 2005, Brazil underwent a long process of preparation, which gained new impetus after it was selected to host some big international events, like the World Cup in 2014 and the Olympic Games in 2016. In an article published in 2021, Fernando Aith, Danielle Rached, and I explain in detail the state of Brazil’s epidemiological legislation when the pandemic hit (Ventura, Aith, Rached, 2021). In essence, we wrote that the 2020 law has essentially the same features as previous legislation. Instead of looking ahead, it establishes norms reactively and in excessive detail. The result is the fragmentation of health law into a plethora of legal and non-legal norms, which can result in inconsistencies and hierarchical conflicts. There also lacks a democratic debate on legal norms pertaining to health, with many key issues remaining unregulated. In other words, the Executive branch should have created more and better regulations for international emergencies. The fact is that there was a lot of resistance in public health quarters in relation to measures like quarantine, lockdown, and mandatory treatment, which explains governments’ hesitation to provide for them in law. But as covid-19 progressed, everyone understood how important it was to address the legal issues related to the pandemic, and not only on a national level, but also internationally. I recall a lot of people asking me what the WHO could do both for us and “against” us.

At that point, in early 2020, my first public statements on the subject were in defense of the Brazilian public health system (Sistema Único de Saúde, SUS, one of the largest public health systems in the world, with decades of existence), especially two aspects. First, based on a study of previous emergencies and the literature on international public health security, I was convinced that true security could only be assured through public health systems (Ventura, 31 jan. 2020). Which meant we had to strengthen SUS. We already had what we needed, but the system had been underfunded, run down, so it needed bolstering immediately before the first case arrived. We had the infrastructure we needed – a large, well-distributed network capable of reproducing protocols throughout the country – and we were in a good position thanks to years of preparation, but we needed a large injection of resources and a new, broad-based appreciation of its professionals. The pandemic was an opportunity that should be seized to immediately change the rates of pay, grant health workers new rights, and tackle precarious employment conditions. Secondly, to my surprise I had the chance to speak on GloboNews television channel (Especialista..., 12 mar. 2020), where I said that as long as the Ministry of Health was in charge of the response, we could feel confident, but that if responsibility for the response shifted to other departments of the federal government, we should be very concerned. Society would have to take the lead, create a scientific committee. State governors would have to take the lead, because if responsibility for the response to the pandemic ceased to be with the Ministry of Health, catastrophe would ensue. A study of past health emergencies shows this to be the case, and I was one of the few people who had done such research from a legal perspective.

A few days after that interview, the organizational structures coordinating the response to the pandemic led by the Ministry of Health were put under the responsibility of a Crisis Committee coordinated by the chief of Staff’s Office. As this committee came under the Executive branch, it had the same status as a ministry. It had 27 members, only two of whom were from the Ministry of Health. A series of executive orders were then passed, granting the new committee its own operations center, and making it the main deliberative body for the covid-19 response. The first paragraph of the study we did for the congressional inquiry, created on April 13, 2021, and installed in the Federal Senate on April 27, 2021, with the aim of investigating the federal government’s actions and inactions in its handling of the covid-19 pandemic, begins with this event, in which the coordination of the Brazilian response to the pandemic is transferred to the chief of Staff’s Office.^2^ I called Fernando Aith, a professor at the Department of Politics, Management, and Health at FSP/USP, and said that Bolsonaro would probably follow the line of Viktor Orban, the prime minister of Hungary, and try to close Congress, under the pretext of containing the disease. At that moment, there was a very strong movement in Brasília against the Federal Supreme Court. February, March 2020 was a key time for the rise of anti-institutional Bolsonarism. I was afraid that this movement would take advantage of the pandemic to introduce exceptionalism, restricting fundamental rights and democracy. What an illusion. I thought Bolsonaro would put the police out on the streets, the Army out on the streets, say that institutions couldn’t function normally due to the emergency, and would even try to use SUS as a trump card. Mandetta, the minister for Health, was already saying that. Until then avowedly against the system, Mandetta began to appear for interviews wearing a SUS vest, saying things like “thank God we have SUS.” Fernando Aith and I concluded that every day we would have to read the official gazettes of the Union and also the states to keep track of what they were doing in legal terms so that we could denounce any potential violations of human rights contained in this legislation, and then, when the pandemic ended, show what vestiges of the regime of exceptionalism remained in the legal system. Fernando recounts this conversation in the documentary *Eles poderiam estar vivos* [They could be alive] (reviewed in this issue of *História, Ciências, Saúde – Manguinhos*), in which we participated a lot. But obviously we didn’t have the money to do that research.

We submitted the research project to Fundação de Amparo à Pesquisa do Estado de São Paulo (Fapesp, one of the country’s biggest research funding agencies) and it responded months later, saying that despite the two opinions pointing to the excellence of the proposal, it was apparently unfeasible, in an evaluation process that even deserved closer investigation. But we didn’t have time for that. We got in touch with Conectas Direitos Humanos (a non-governmental human rights organization with a Global South perspective), which immediately understood what was at stake and offered us four excellent interns. Rossana Reis, at the time a professor at the Department of Political Science at USP, and the international relations expert Camila Asano, from Conectas, joined us as research coordinators. We created a qualitative evaluation form for each legal norm. I didn’t want a robot, I didn’t want something just quantitative; I wanted people to read each norm and identify the type of norm, the executing authority, the issue involved, the potential for the curtailment of rights, the potential for vulnerable groups to be affected. We made a database of these norms. We trained the team for this task, and they passed on everything they identified to us. We decided to create a bulletin, *Direitos na Pandemia* (Rights in the Pandemic), to publicize the partial results of the study, which was also financed by Conectas, and we quickly realized that the government was contributing to the spread of the virus. We were facing a different, more complex type of authoritarianism. We also realized that Brazilian society believed in the thesis of the double-faced strategy – which was later reinforced in the deposition of the then minister for Health, Eduardo Pazuello, in the congressional inquiry – namely, the idea that the president would talk all sorts of nonsense on the internet, but that actually his actions were different, that he fought the disease, despite his limitations. This was a thesis that prevailed throughout 2020: that the president was irresponsible and incompetent, spoke nonsense to his supporters, but said one thing and did something else. But what we claim is that, on the contrary, he was doing exactly what he said he was doing. Of course there was resistance to what he did, but that’s another matter. Some institutions resisted. But he was being coherent and there was a state strategy. The first person to publish our thesis, which for us is not a thesis, but reality, was Eliane Brum (22 jul. 2020), a fabulous award-winning journalist, who had the courage to publish an interview in *El País Brasil* in which I said that a crime against humanity was taking place in Brazil. Gilmar Mendes, the renowned jurist and minister of the federal supreme court, had already spoken explicitly about genocide. But we were both the target of strong criticism and written off as militants.

It was only when those horrific scenes were seen in Manaus in mid-January 2021, when the capital of the state of Amazonas – its hospitals full to overflowing and out of oxygen – became the epicenter of the covid-19 pandemic, that we stopped being seen as militants and started to be recognized as researchers. We thought about how we could present the results of our research, because it’s very difficult to get people interested in legal norms, written in a very restricted jargon, whose effects could be obscure. We decided to make a timeline with the following classifications: “government actions,” “legal acts,” and “anti-public health propaganda.” Even someone who wasn’t particularly interested in presidential vetoes, directives, or executive orders could see how these norms lined up with Bolsonaro’s statements and government decisions. Only then did we manage to translate our research into a format that could be understood,^3^ which had huge repercussions. Senators keen to hold an inquiry into the handling of the pandemic, like Randolfe Rodrigues, Humberto Costa, and Alessandro Vieira, got in touch and asked us for more details about the study.^4^ The congressional inquiry commissioned us to update the study, which we delivered in May 2021. The study was also translated into English thanks to professor Octavio Ferraz, from the Transnational Law Institute at King’s College London (see Ventura et al., 5 ago 2021). Even so, it’s no more than a summary, because we actually found thousands and thousands of norms and orders in the official gazette [*Diário Oficial*]. Conectas stopped our funding in 2021 because it felt that the initiative had already served its purpose, and we started working with the National Council of Health Secretaries to consolidate and expand the database. Essentially, the initial aim of our study was to follow the evolution of the Brazilian legal system, especially human rights violations, thinking that there would be a point of closure. But what we found was an ultraliberal idea, which could be called epidemiological neoliberalism, which simply means letting people die. I believe this is one of the things that makes Brazil’s response to the pandemic so unique.

*Carlos Henrique Assunção Paiva: Thank you, Deisy. Now I’d like to ask the following question. Nobody can say that the covid-19 pandemic came out of the blue. Several international agencies were already contemplating such a possibility, including the possible emergence of a coronavirus with pandemic potential, right? Given this, how would you evaluate the overall international response to the covid-19 pandemic?*

I always joke that I’m the only person who reads the WHO documents. I read everything. But seriously, that’s a very important question. There are plenty of documents on preparations for health emergencies. Actually, there’s even an International Health Regulation on it, which was passed in 2005 and is in force in 196 states. We may not always agree with WHO recommendations, but there’s a structure that is constantly issuing new standards, scientific evidence, recommendations, whether or not we agree with them all, and the fact is that it also provides reliable standards and guidance for emergencies. The WHO wasn’t the only international organization to have prepared in detail for episodes like this. The World Bank, for example, has a pandemic preparedness fund. I don’t agree with that approach, because it’s based on insurance, a large pandemic insurance market, as Felix Stein and Devi Sridhar (2017) have shown. But the fact is that they exist, and they existed many years before. It’s quite misleading to say that international organizations didn’t warn of the imminence of an emergency of this type. But having sounded the warning, it’s important to think about what these organizations were able to do in response to the *fait accompli*. These are two very different things. It’s one thing to offer means for preparedness with which we may or may not agree; it’s quite another to establish the limits of these international organizations when an event of this type takes place, because warning is not enough. When something happens, international organizations, and particularly the WHO, have a role to play.

In this pandemic something important happened. Compared with the 2014-2015 Ebola crisis, there was a new appreciation of the WHO. In the Ebola crisis, the international community and particularly the US president at the time, Barack Obama (2009 to 2017), didn’t include the WHO, didn’t see it as capable of coordinating that response. A United Nations public health mission (United Nations Mission for Ebola Emergency Response) was created, which coordinated the international action, received funding, and took the lead in the response, under the auspices of the UN Security Council. The WHO served as technical advisor to the mission, which acted at the epicenter of the crisis in Central Africa, mainly Liberia, Sierra Leone, and Guinea, with thousands of US soldiers, together with the representative of the UN Secretary General, at the time Ban Ki-moon, and the strong leadership of Obama to coordinate the response by the UN through this mission. At the same time, Donald Trump was gaining power inside the USA (president from 2017 to 2021), attacking Obama strongly and accusing him of letting ebola into the country after a US citizen with the disease was repatriated. Ebola was an important instrument of political manipulation by the far right. But having health emergencies handled by the UN Security Council would be a very bad sign, signaling the securitization of public health issues (Ventura, 2016). That means treating health as an international security issue, looking more at containing the spread of the disease to the developed world than at its effects on the affected populations.

Then came the emergence of congenital zika syndrome (CZS) in 2015 and 2016, whose epicenter was Brazil, with completely different characteristics from ebola. The WHO came here and praised Brazil’s work. Not only did the Brazilian government agree with declaring CZS an international emergency as a way to strengthen the response strategy domestically, but there was also the issue of the Olympics and Paralympics in the country, which was decisive for declaring the emergency. That’s why it’s hard to compare. But when a phenomenon as big as covid-19 comes along, it’s very important for the WHO to be at the center of the international response. That’s one positive element in the analysis. The WHO managed to coordinate the response and managed to take the lead. This resulted in attacks, to the point of the US leaving the WHO. Trump announced the country’s departure, then Joe Biden reversed it when he took office in January 2021. It’s incredible that this actually happened. Historically – and you know this much better than I do – the other withdrawal occurred between 1949 and 1955, when Soviet countries left because they believed the WHO would be pro-US. It was a Brazilian, Marcolino Candau, the director-general of the WHO at the time, who brought them back. But it’s incredible that the Soviets left for that reason decades ago and that the US, facing covid-19, renounced the leading role it had earned and withdrew from the WHO. Brazil’s far-right government has also criticized the WHO, as have other far-right governments.

It cannot be denied how important a role the organization played in the response to the pandemic, but it has enormous limits, which could be summed up as an issue of funding – a chronic problem for the organization. What I mean is, the WHO has limited resources, and these are subject to the donors’ priorities. There’s a second problem, which is the regulatory limits of its actions, because WHO recommendations are not mandatory, and its mechanisms for enforcing state compliance are fragile, consisting essentially of self-assessments. These two elements – funding and the absence of more effective enforcement mechanisms – could be summed up in a single sentence: a lack of political will on the part of the states to give the WHO greater capacity to act. In a scenario other than the rise of the far right around the world and the retreat of multilateralism, the natural thing would be for states to make the WHO stronger, but that’s not the reality now, when we’re seeing a retraction of multilateralism. There wasn’t the kind of political strength needed to ensure the WHO had more resources so it could set its priorities more freely and also impose greater constraints on states to comply with its recommendations. Added to which there’s perhaps the most obvious aspect of the WHO’s inability to cope with an emergency of this type: access to vaccines. Finally, the WHO’s director-general stated that intellectual property rights should be made more flexible during a pandemic. Its director-general, Tedros Adhanom, spoke of “vaccine apartheid” to describe the irrational hoarding of vaccines by rich countries. It’s very strong wording for an international functionary to use, as this kind of vocabulary isn’t usually used in such circles. As well as a reference to the history of South Africa, apartheid is listed as a crime against humanity in the Rome Statute, the treaty that established the International Criminal Court, based in The Hague (Netherlands), in 1998. But Tedros didn’t have enough political clout. What he did manage to do, for now, was to create a negotiating body for an international agreement on pandemics.

Supposedly, this agreement will contain provisions on intellectual property, but from the positions the member states are taking, it doesn’t look as if we’ll manage to effectively ease this in the context of international law. Brazilian leadership is greatly needed, because in previous years, like in 2001, with the Doha Declaration, which recognized the importance of generic antiretrovirals, Brazil, India, and other countries managed to get recognition that exceptions to intellectual property rights can be justified for public health purposes. With the change of government in Brazil, the country must take the lead in this agenda, bring back the Doha principles and try to put this treaty on pandemics in order. I think this should be Brazil’s overarching position, to articulate the Global South in this respect.

To my mind, the international response has been compromised by the limits states have imposed on the WHO. One explanation for the lack of political will to strengthen the WHO could be sovereigntism on the part of the member states: the idea that they don’t want to yield any of their competences to international organisms. But my view is that there’s something stronger than that, something simpler. When you read the International Health Regulations – and very few people in the world have read them – you conclude that the only way to truly fulfill those commitments is by means of public health systems with universal access, which meet the needs of regulation, public policies, borders, human resources, risk communication, laboratory networks, etc. Where do we have these? In large public health systems. The response to a pandemic or any other international emergency will only be effective when there’s universal access to health, and that’s a problem because there are political forces acting in the opposite direction at every level. During an emergency, the WHO will do what it just did: it will make recommendations, it will point out when states aren’t being efficient in their response, but it will do so with great care, because as an international organization it depends on its member states. Even so, in the case of Brazil, in several press conferences, the WHO noted that it was concerned about the country, that herd immunity by contagion was ethically unacceptable and scientifically baseless. In future emergencies, if the regulations don’t change, the WHO will continue to try to coordinate collaboration initiatives such as Covax, which the WHO created to ensure the equitable distribution of covid-19 vaccines, but they’re limited by the interests of certain states. The question is: how can we avoid new emergencies? And that raises two big issues: the one I mentioned earlier, of national health systems, and the environmental issue. The environmental origins of health emergencies are evident, such as the invasion of natural habitats, in the case of ebola; rudimentary sanitation, in the case of CZS; or else intensive livestock farming, in the case of A(H1N1) influenza, which started in pig farms in the USA and Mexico. In the Global Health and Sustainability Program at FSP/USP, we’re focusing on understanding the interface between environmental and global health issues, which is enormous and complex. Going back to the criticisms leveled at the WHO, they’re very superficial because they don’t take account of how the organization works, which is linked to the member states’ positions. They don’t take account of this very difficult political time, of retreat from multilateralism, and similarly they don’t take account of our need for public investment in health and a change in political bias in the perception of health to control emergencies. Otherwise, we’ll just be chasing our tails every time a new emergency comes along.

As for the International Health Regulations, very few people work on this issue with a critical perspective. These regulations are currently under review, and rich countries seem to be taking the following stance: “let’s focus on being able to identify whenever something in the poor world might get into the rich world, so we can close our borders quickly.” In the international context we’re living in, there’s a concrete, tangible risk associated with putting faith in systems for surveillance, in the security sense of the term, rather than strengthening national response capacities. Security can have an emancipatory meaning. When we say that SUS is fundamental for the security of the Brazilian population, we’re using the word “security” in the emancipatory sense of protecting life, but when the US and other developed countries focus on detection methods and not on assuring health for all, the word “security” doesn’t necessarily have an emancipatory meaning. It often simply corresponds to the idea that the poor are a threat to the rich.

*Carlos Henrique Assunção Paiva: I’d like to draw attention to the feat, in the Brazilian context, of creating a universal public health system in very adverse international circumstances, during the administrations of Ronald Reagan and Margaret Thatcher. From an ideological, political, and economic viewpoint, it was a very hostile international scenario. It was all very difficult, and we have to recognize that SUS has problems, so to speak, in its DNA, starting with an issue that Deisy raised, which is funding. The funding it receives isn’t remotely compatible with providing universal health care for a country of this size. So, if we now focus in on the domestic setting, how can we assess the state of this public health system in a very specific context? Could you look specifically at this Brazilian health system and think about how it responded and how we can judge it, for example, in view of federative issues – something else that is far from new, since it’s already been a part of the scope of SUS for a long time. Deisy, I have the impression that the pandemic has allowed us to see more vividly some chronic preexisting problems in the functioning of SUS.*

Overall, I would say that SUS picks up the pieces of our economic system. What I mean is, I see SUS as one of the only systems of redistributive justice Brazil has ever had. For me, SUS is the jewel in the crown of Brazilian democracy. I’m talking about material democracy, access to health care. A person may have no money to pay for treatment, but they won’t die without care. Vaccination programs, public health policies, primary health care, with all the shortcomings these programs may have, are all material democracy. Limitations aside, they’re still redistribution. At the same time as being an expression of democracy and the enshrinement of rights, this system enables a very violent economic order to be maintained, because it mitigates its harmful effects on people’s physical and mental wellbeing. SUS works like a shock absorber for the brutal, feral capitalism that has taken root in Brazil, associated with other forms of authoritarianism, which under Bolsonaro was taken to the extreme of outright plunder. They simply looted the public coffers, not only through corruption, but also through the misuse of public resources.

The same was the case with the covid-19 pandemic. I believe the federal government had this in its sights and envisaged the perfect crime, knowing that SUS would prevent millions of deaths. It mistakenly decided on an “acceptable” number of deaths. They knew that in an election year, governors and, above all, mayors would not want Army trucks full of dead bodies or mass graves. But sadly that’s exactly what happened, albeit to a much lesser extent than would have been the case in the absence of SUS. There are cases that need to be studied. Something that fascinates me, for example, is the response seen in Santa Catarina, a state that’s overwhelmingly pro-Bolsonaro. I read a report that talked about a “curfew” in Santa Catarina. Then the state government denied it: “no, no, there’s no curfew here. Here, the only times you can’t go out are between midnight and five in the morning, but there’s no curfew” (Caldas, 6 dez. 2020). That is, some states and municipalities actually experienced this contradiction between alignment with the federal government and the need to avoid even worse numbers and situations, like what happened in Manaus.

Manaus epitomized what was being done. But I’m sure that in its calculations, the federal government counted on mayors not letting people die and opening more ICU beds so things wouldn’t reach the point of infamy. But we reached a level of infamy in Manaus, when for a few weeks Brazil could see what was happening. I always say that we took a lot of hits and were called militants, as if our work wasn’t scientific, as if what we were saying was unfounded. But one of the happiest days of my life and of my career was when I saw a TV presenter say that they didn’t need a study by a research group at USP to demonstrate that the government was spreading the virus. At that point, we realized we’d done our job. But then the issue fell into oblivion and ended up almost completely absent from the election campaign. Bolsonaro’s criminal behavior was somehow taken for granted. I see SUS as a great democratic asset, but because of the limits imposed on it, it also serves as a buffer for this structural violence.

*Claudia Agostoni: Everything you’re saying is fascinating even for me, living in Mexico. Have you compared the international collaboration or the international responses with other countries in Latin America or the Southern Cone, like Paraguay and Argentina? Here in Mexico, we had a response, or rather a non-response, as well as the spread of the virus, disputes between the federal government and the states, concealment of numbers, and the neoliberal circulation of the virus about which you spoke. I would like to know if you have seen other similar circumstances.*

Sadly not. I’d love to, but we didn’t have the means to make comparisons. I particularly want to study Mexico. In the past, I studied the Mexican response to H1N1 influenza, but I haven’t spoken about Mexico in the context of covid-19 precisely because we haven’t yet studied this case in depth. If any partner is willing to carry out a comparative study with Brazil, we’re very interested.

*Carlos Henrique Assunção Paiva: One thing that attracted my attention is how historians were called upon, together with scientists, by the so-called mainstream media, among others, to try to provide some historical parameters for an event that seemed completely new. Even I, who had never been a scholar of pandemics, found myself in the challenging position of trying to gain familiarity with the subject and making comparisons so we could think a little about our public memory of other experiences. Historians were called on more at the beginning, when there were so many uncertainties. But from now on, as we enter the third year of the pandemic, what role do you think historians will have in this dialogue with the collective memory of the pandemic?*

Your question is an important part of my research agenda. I have argued that the pandemic is about memory, truth, and justice. It’s an elementary question for anyone with a background in international law and who has studied war crimes and crimes against humanity, and even more so for anyone who works with international law from a human rights perspective, like me. This background was crucial for the perception we had of the pandemic, and especially for my involvement in the struggle to hold the military accountable for the serious human rights violations committed in Brazil during the military regime (1964-1985). I learned a lot with the lawyer Paulo Abrão and a host of other extraordinary legal experts with whom I worked, especially between 2008 and 2014, when the National Truth Commission was created, later led by the lawyer Pedro Dallari. I think the fact that we had studied crimes against humanity, state-sponsored violations, was the reason why our gaze was so attuned to picking up the historical dimension of what was happening. For example, identifying the idea of a systematic plan against a civilian population, which is a prerequisite for a crime against humanity. A systematic plan against a civilian population is not just about having firing squads; it’s also about spreading false information that incites people to expose themselves and others to risk. There are precedents for this in international jurisprudence resulting from the interpretations of dramatic episodes in recent history, such as the role of Radio Mille Collines in inciting the population to violence in the Rwandan genocide in 1993 and 1994.

For all these reasons, I have no doubt that covid-19 in Brazil must be treated this way, with memory policies. We need instruments to tell us what happened, to give victims a voice. There are at least three covid-19 victims associations, which are trying to fight for some recognition. These associations were completely silenced during the election campaign. And our report for the congressional inquiry, a very important instrument of memory, but whose political shield prevented greater responsibility from being attributed. However, these are crimes that have no statute of limitations. A day may come when the circumstances are more favorable, and the evidence will have to be there. So, a policy of memory and truth is needed so that justice can be done at a later stage.

We had trouble prosecuting these crimes within the Brazil legal system, but we could look to international jurisdictions. There’s the International Criminal Court. This quest for truth must be taken on. We also have reparation mechanisms, because people who died as a result of state action or inaction must be indemnified. There are already several lawsuits. It’s a very slow process, but it’s a process that must be supported, encouraged, and made known. It’s part of what is called transitional justice. In a debate with some fellow foreign legal experts, they said regime change needs to take place before there can be transitional justice. I’m not saying we’re going to achieve transitional justice for the pandemic. What I’m saying is that we’re going to make use of mechanisms that have been created for transitional justice, which we already know from other historical processes, and which can actually be adapted to fit the motto of transitional justice: “never forget so it can never happen again.” Without policies for memory, truth, and justice regarding covid-19, the next pandemic will be much worse. There’s no doubt in my mind as to how decisive a role historians play in consolidating this memory, which is very fragile. People have already forgotten the pandemic. It was already a marginal issue in the election campaign, when it should have been the most important one. When Bolsonaro appeared on *Jornal Nacional*, on TV Globo, in the series of interviews with presidential candidates, it was one of the saddest days of my life, because the pandemic was taken as a given. He was on one of the most important Brazilian television news shows in a live broadcast and nobody asked him. Someone should have said: “you spread covid-19 in Brazil.” He wasn’t held to account.

Eduardo Pazuello, the disastrous minister of Health between 2020 and 2021, was one of the candidates who received most votes in the October 2022 elections! That just goes to show how much we need memory, truth, and justice. Remember, during the congressional inquiry, Pazuello said something really important: that the president’s discourse on the internet was one thing, but his state policies were different. That’s just not true: they were the same policy. If historians of today and tomorrow buy into this thesis that Brazilian state action during the pandemic was no more than propaganda, we will consolidate something that wasn’t true. You have to look at the documents. I think the role of history is fundamental, and what we’ve tried to do is preserve all these instruments so that historians can do their work.

*Claudia Agostoni: The question of forgetting, of collective amnesia, is surprising, because the pandemic isn’t over yet. I think that as historians, we have a lot to say about this topic in particular. But forgetting, the act of ignoring, of not being there anymore, is a problem we’ve seen many times.*

*Carlos Henrique Assunção Paiva: I suspect that the Bolsonaro government really intended to erase memory, but the pandemic has a latent, diffuse memory, even if it’s badly organized in collective terms. But what strikes me is that if Bolsonaro had listened to the experts from SUS and let them do what they know best, he would have won the election in the first round. I have no doubt about that, even with all the outrageous behavior he’s known for. Instead, he invested in ultraliberalism, which resulted in a huge amount of collective pain, which has not yet been recorded in full. And despite everything he did, he still got to the run-off with Lula in a context of very strong anti-Worker’s Party [to which Lula is affiliated] sentiment. There’s so much yet to assess.*

The congressional inquiry played an essential role. Some say it came to nothing, but that’s not true. The congressional inquiry kept the pandemic on the agenda in parliament and in the media for months, when it was already starting to be forgotten. It gave voice to witnesses, victims, and families. It compiled evidence and produced new evidence. And above all, it treated what happened in Brazil for what it actually was: a set of crimes that resulted in the preventable death of hundreds of thousands of people, which need to be investigated, prosecuted, and judged and should never happen again.

*Marcos Cueto: Thank you very much, Deisy. Your comments are very important and valuable for us and for the readers of História, Ciências, Saúde – Manguinhos.*

## NOTES

1 A version in Spanish of my thesis, published by the Konrad Adenauer Foundation’s office in Uruguay, is available on the website of the Universidad Nacional Autónoma de México (see Ventura, 2004).

2 The study *A linha de tempo da estratégia federal de disseminação da covid-19* [Timeline of the Federal Strategy for the spread of covid-19] was prepared as part of the research project Mapping and Analysis of the Legal Norms for Covid-19 Response in Brazil, run by Cepedisa/FSP/USP, and commissioned by the congressional inquiry created by the Federal Senate in 2021 (Cepedisa/FSP/USP, 28 maio 2021).

3 It was published for the first time in January 2021. See: Ventura, Reis (2021).

4 In their book on the workings of the congressional inquiry, the senators Randolfe Rodrigues and Humberto Costa acknowledge how the study served as inspiration for the inquiry (Costa, Rodrigues, 2022, p.31-32).
